# Screening for *Arabidopsis* mutants with altered Ca^2+^ signal response using aequorin-based Ca^2+^ reporter system

**DOI:** 10.1016/j.xpro.2021.100558

**Published:** 2021-05-23

**Authors:** Shujing Sun, Xiaoyan Zhang, Kong Chen, Xiaohong Zhu, Yang Zhao

**Affiliations:** 1 Shanghai Center for Plant Stress Biology, and CAS Center for Excellence in Molecular Plant Sciences, Chinese Academy of Sciences, Shanghai 200032, China; 2 State Key Laboratory of Crop Stress Adaptation and Improvement, School of Life Sciences, Henan University, Kaifeng 475001, China; 3 State Key Laboratory of Cotton Biology, School of Life Sciences, Henan University, Kaifeng 475001, China; 4 University of Chinese Academy of Sciences, Beijing 100049, China

**Keywords:** Genetics, High Throughput Screening, Model Organisms, Molecular/Chemical Probes, Plant sciences

## Abstract

Environmental stimuli evoke transient increases of the cytosolic Ca^2+^ level. To identify upstream components of Ca^2+^ signaling, we have optimized two forward genetic screening systems based on Ca^2+^ reporter aequorin. AEQ*sig6* and AEQ*ub* plants were used for generating ethyl methanesulfonate (EMS)-mutagenized libraries. The AEQ*sig6* EMS-mutagenized library was preferably used to screen the mutants with reduced Ca^2+^ signal response due to its high effectiveness, while the AEQ*ub* EMS-mutagenized library was used for screening of the mutants with altered Ca^2+^ signal response.

For complete details on the use and execution of this protocol, please refer to Chen et al. (2020) and Zhu et al. (2013).

## Highlights

• Highly efficient systems for screening of Ca^2+^ signal mutants in *Arabidopsis*
• Step-by-step instructions to analyze Ca^2+^ signal response using Ca^2+^ reporter aequorin
• AEQ*sig6* system for identifying mutants impaired in shoot-based Ca^2+^ signaling
• FAS system for isolating tissue- or stimuli-specific Ca^2+^ responsive mutants

## Graphical Abstract

**Figure:**
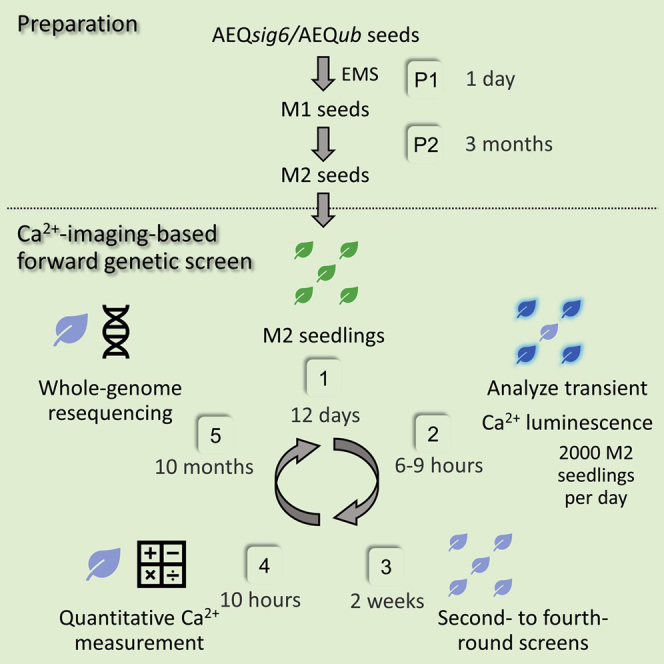

## Before you begin

Screening of *Arabidopsis* mutants with altered Ca^2+^ signal response using the AEQ-based Ca^2+^ reporter system (Col-0 wild type expressing *35S* promoter-driven *apoaequorin*) has led to the identification of receptors or regulators involved in Ca^2+^ signaling in response to environmental changes during the past six years (Table 1). The Ca^2+^ signal is transiently induced by multiple stimuli, including environmental stresses such as osmotic, salt, cold, heat, wounding, and small molecules such as heavy metal ions, H_2_O_2_, ATP, HCO_3_^−^, amino acids, flagellin, and quinones (Table 2). However, there may exist unidentified stimuli that induce Ca^2+^ signal. Any given stimuli should be tested for the strength of Ca^2+^ response using either the AEQ*sig6* (cotyledon-albino mutant expressing *apoaequorin*) or AEQ*ub* (Col-0 wild type expressing *ubiquitin* promoter-driven *apoaequorin*) seedlings before screening for Ca^2+^ responsive mutants.

***Note:*** Given the aequorin luminescence peak (465 nm) falls within the absorption spectrum of chlorophyll *b*, the use of AEQ*sig6* seedlings minimizes the interference of chlorophyll *b* due to its albino cotyledons. As expected, the AEQ*sig6* seedling has enhanced Ca^2+^ luminescence signal response compared with the wild-type AEQ*35S* and AEQ*ub* seedling (Chen et al., 2020). Therefore, the AEQ*sig6* EMS library is an EMS mutagenized pool optimized to screen the mutants with reduced Ca^2+^ signal response. Alternatively, the AEQ*ub* EMS library is considered a better choice than the AEQ*35S* EMS library because *35S* promoter-driven *apoaequorin* is often silenced when crossed with T-DNA insertion mutant or transformed with *35S* promoter-driven transgenes for gene functional analysis, most likely due to siRNA-mediated transcriptional silencing (Mlotshwa et al., 2010). Therefore, we recommend an AEQ*ub* EMS library or a genetic background of interest expressing an AEQ*ub* construct for the screening of the mutants with altered Ca^2+^ signal response. This protocol aims to screen mutants with altered Ca^2+^ signals using Aequorin-based luminescence imaging. Screening Ca^2+^ responsive mutants with the plate reader can be conducted by following two other protocols (Tanaka et al., 2013, Mittal et al., 2020).

**Table 1:** Isolated mutants with abnormal Ca^2+^ signal response

Mutant name	The phenotype of the mutants	Gene description	References
*hpca1/card1*	Reduced extracellular H_2_O_2_-induced and DMBQ-induced Ca^2+^ increase; insensitive to H_2_O_2_-induced stomatal closure and unresponsive to DMBQ	Leucine-rich-repeat receptor kinase	(Wu et al., 2020, Laohavisit et al., 2020)
*lore*	Reduced extracellular LPS-induced Ca^2+^ increase; LPS-insensitive	Lectin S-domain receptor kinase	(Ranf et al., 2015)
*dorn1*	Reduced extracellular ATP-induced Ca^2+^ increase	Lectin receptor kinase	(Choi et al., 2014)
*moca1*	Reduced salt stress-induced Ca^2+^ increase; hypersensitive to salt stress	Glucuronosyltransferase	(Jiang et al., 2019)
*bon1*	Reduced hyperosmotic stress-induced Ca^2+^ increase; irresponsive to osmotic stress in ABA accumulation and gene expression	Ca^2+^-responsive phospholipid-binding protein	(Chen et al., 2020)
*osca1*	Reduced hyperosmotic stress-induced Ca^2+^ increase	Calcium channel	(Yuan et al., 2014)
*cngc2/4*	Reduced PAMP-induced Ca^2+^ increase	Calcium channels	(Tian et al., 2019)
*arp2*	Increased salt stress-induced Ca^2+^ increase	Actin-binding protein	(Zhao et al., 2013a)
*qua1*	Increased salt stress-induced Ca^2+^ increase	Glycosyltransferase	(Zheng et al., 2017)
*mci1*	Reduced responses to HCO_3_^−^/CO_2_ for [Ca^2+^]_cyt_ changes and stomatal movement	Unknown	(Tang et al., 2020)
*mcs1*	Enhanced responses to HCO_3_^−^/CO_2_ for [Ca^2+^]_cyt_ changes and stomatal movement	Unknown	(Tang et al., 2020)

**Table 2:** The recommended ranges for stimuli

Stimuli	Recommended concentrations	References
**Environmental factors**		

Gravity		(Toyota et al., 2008)
Circadian		(Love et al., 2004, Martí Ruiz et al., 2018)
Light		(Harada et al., 2003, Martí Ruiz et al., 2018, Xu et al., 2007, Baum et al., 1999)
Dark		(Martí Ruiz et al., 2020)

**Stresses**		

Hyperosmotic stress	200–600 mM mannitol, sorbitol, glucose, or sucrose	(Knight et al., 1997, Yuan et al., 2014, De Vriese et al., 2019)
NaCl	75–200 mM NaCl	(Knight et al., 1997, Pan et al., 2012, Jiang et al., 2019, Cao et al., 2017)
Cold	0°C–10°C	(Knight et al., 1996, Mori et al., 2018)
Heat	37°C–45°C	(Zhu et al., 2013, Wang et al., 2015)
Anoxia		(Sedbrook et al., 1996)
pH	3.5–4.5	(Zhu et al., 2013, Russell et al., 1996)
Mechanical perturbation	Touch	(Russell et al., 1996, Monshausen et al., 2009, Matthus et al., 2019)
Wounding	Cropping	(Vincent et al., 2017, Toyota et al., 2018)

**Secondary messengers and chemicals**		

H_2_O_2_	1–5 mM H_2_O_2_	(Zhu et al., 2013, Wu et al., 2020)
cGMP	10 μM	(Volotovski et al., 1998)
cAMP	10 μM	(Volotovski et al., 1998)
GSH	10–1000 μM	(Li et al., 2013, Qi et al., 2006)
GSSG	1000 μM	(Qi et al., 2006)
Nicotinamide	50 mM	(Abdul-Awal et al., 2016, Pétriacq et al., 2016)
NAD^+^	0.1–5 mM	(Pétriacq et al., 2016)
NADP^+^	0.1–5 mM	(Pétriacq et al., 2016)
NADH	0.1–5 mM	(Pétriacq et al., 2016)
NADPH	0.1–5 mM	(Pétriacq et al., 2016)
SNP	5 μM–10 mM	(Abdul-Awal et al., 2016, Aboul-Soud et al., 2009)
SNAP	5 μM–500 μM	(Abdul-Awal et al., 2016)
Methanol	0.1%–5%	(Tran et al., 2018)

**Pathogen-associated molecular patterns (PAMPs)**		

Flagellin	0.125–2 μM	(Ma et al., 2012, Zhu et al., 2013, Cao et al., 2017)

**Damage-associated molecular patterns (DAMPs)**		

Plant elicitor peptides	20 nM–4 μM Pep1/3	(Ma et al., 2012, Cao et al., 2017, Qi et al., 2010)
Nucleotides	10–1000 μM ATP/GTP	(Choi et al., 2014, De Vriese et al., 2019, Tanaka et al., 2010)
L-Glu	100 μM–10 mM	(Li et al., 2013, Zhu et al., 2013)
L-Gly	100 μM–10 mM	(Li et al., 2013, Zhu et al., 2013)
Cys	100 μM–10 mM	(Li et al., 2013, Zhu et al., 2013)
L-Ser	100 μM–10 mM	(Li et al., 2013, Zhu et al., 2013)
L-Ala	100 μM–10 mM	(Li et al., 2013, Zhu et al., 2013)
L-Asp	100 μM	(Li et al., 2013)
L-Asn	100 μM–10 mM	(Li et al., 2013, Zhu et al., 2013)

**Hormones**		

JA	450–900 μM	(Zhu et al., 2013)
SA	180 μM–5 mM	(Zhu et al., 2013, Kawano et al., 1998, De Vriese et al., 2019)
Auxin	167–500 μM IAA/NAA/2,4-D	(De Vriese et al., 2019)
Gibberellin	10–500 μM GA_3_/GA_4_	(De Vriese et al., 2019, Okada et al., 2017)
Cytokinin	167–500 μM BAP	(De Vriese et al., 2019)
Brassinolide	100 mM eBL	(Zhao et al., 2013b)

**Heavy metals**		

Cu^2+^	10 mM CuCl_2_	(Zhu et al., 2013)
Cd^2+^	10 mM CdCl_2_	(Zhu et al., 2013)

### Generating the ethyl methanesulfonate (EMS)-mutagenized mutant pool

**Timing: 12 weeks**

1. Soak 0.5–1 mL (17500–35000 seeds) of the AEQ*sig6* or AEQ*ub* seeds in dH_2_O overnight (9–11 h) in a 50 mL Falcon tube.
2. Discard dH_2_O and treat the seeds with 20 mL 0.4% EMS solution in 100 mM potassium phosphate buffer (pH7.5) (Kim et al., 2006).

**CRITICAL:** EMS is extremely toxic. Handle EMS with gloves in a chemical fume hood. Leave EMS contaminated water and labware in a beaker containing 1 mol/L NaOH for at least one week in the hood with warning labels for decontamination.

3. Seal the tube well with parafilm, and put it into a zip-lock bag with paper towels and warning labels.
4. Gently shake the tube for 8 h at room temperature (20°C–25°C).
5. Place the tube vertically and pipette off the EMS solution after the seeds settled.
6. Wash the seeds with dH_2_O about 20 times.
7. Plant the M1 seeds with about 0.3 cm apart on 0.3% Phytagel containing 1/2 MS medium nutrients (PhytoTech), 1% sucrose, pH5.7.
8. Transfer the eight-day-old seedlings to the soil at a density of 16 seedlings per pot.
9. Grow the seedlings on optimized growth conditions to avoid a stressed environment.
10. Harvest the M2 seeds from each pot as an individual pool.

**CRITICAL:** A small pool allows us to regain candidate mutants with low vitality that may be lost due to growth defect, given that EMS often causes multiple random mutations that affect plant growth and fertility. Therefore, we suggest a single pedigree-based seed collection as previously described (Mittal et al., 2020), although it is labor-intensive.

***Note:*** To avoid poor seed vigor or growth defect of plant materials, the EMS mutagenized pool should be generated freshly from the plants under non-stress conditions. The M1 seedlings are grown under an isolative space to avoid cross-pollination.

## Key resources table

**Table:** 

REAGENT or RESOURCE	SOURCE	IDENTIFIER
**Chemicals, peptides, and recombinant proteins**		

Ethyl methanesulfonate (EMS)	Sigma-Aldrich	CAS# 594-43-4
Potassium phosphate buffer (pH 7.5), 0.5 M, pH7.5	TIANDZ	CAS# 25-05504
NaOH	Sinopharm Chemical Reagent	CAS# 1310-73-2
Sodium hypochlorite (NaClO)	Sinopharm Chemical Reagent	CAS# 7681-52-9
Murashige & Skoog (MS) Basal Salt Mixture	PhytoTechnology	Cat#M524
Sucrose	Sinopharm Chemical Reagent	CAS# 57-50-1
Phytagel	Sigma-Aldrich	CAS# 71010-52-1
Agar	Sigma-Aldrich	CAS# 9002-18-0
Ethanol	Sigma-Aldrich	CAS# 64-17-5
Triton X-100	Sigma-Aldrich	CAS# 9002-93-1
Mannitol	Sigma-Aldrich	CAS# 69-65-8
H_2_O_2_	Sigma-Aldrich	CAS# 7722-84-1
NaCl	Sinopharm Chemical Reagent	CAS# 7647-14-5
CaCl_2_.2H_2_O	Sigma-Aldrich	CAS# 10035-04-8
Cefotaxime	Sigma-Aldrich	CAS# 64485-93-4
HgCl_2_	Sigma-Aldrich	CAS# 7487-94-7
aCoelenterazine h	Nano Light	CAS# 50909-86-9

**Experimental models: Organisms/strains**		

*Arabidopsis*: Col-0	Widely distributed	N/A
*Arabidopsis*: *bon1-7* (*osmo1-1*)	(Chen et al., 2020)	N/A
*Arabidopsis*: AEQ*35S*	(Knight et al., 1991)	N/A
*Arabidopsis*: AEQ*ub*	(Zhu et al., 2013)	N/A
*Arabidopsis*: AEQ*sig6*	(Chen et al., 2020)	N/A

**Recombinant DNA**		

pMAQ	(Knight et al., 1991)	N/A

**Software**		

WinView/32	Roper	N/A

**Other**		

Cooled charge-coupled device	Princeton Instruments, New Jersey, USA	N/A
Luminometer	GloMax, Promega	N/A
Microplate reader	Molecular Devices	SpectraMax i3x
Percival incubator	Percival	CU36L5
Adhesive PCR Plate Seals	Thermo Scientific	AB0558

^a^ Critical reagent

## Materials and equipment

A cooled charge-coupled device (CCD, Princeton Instruments, New Jersey, USA) controlled by WinView/32 (Roper) is used to acquire Ca^2+^ luminescence images. The optimal working temperature for the CCD camera is −100°C. The CCD imaging system should be placed in a dark room to avoid the interference of light. The equivalent imaging system can be used for the same purpose. Also, a luminometer (GLOMAX, Promega) or Microplate Reader (SpectraMax i3x, Molecular Devices) with or without an auto-pump injector system can be used for quantitative measurement of Ca^2+^ signal dynamics.

**Table:** EMS working solution

Reagent	Final concentration	Amount
Ethyl methanesulfonate	0.4% v/v	80 μL
Potassium phosphate buffer (pH 7.5), 0.5 M	100 mM	4 mL
ddH_2_O	n/a	16 mL
**Total**	**n/a**	**20 mL**

Prepare the working solution on the day for sample processing.

**CRITICAL:** EMS is extremely toxic. Handle EMS with gloves in a chemical fume hood. Leave EMS contaminated water and labware in a beaker containing 1 mol/L NaOH for at least one week in the hood with warning labels for decontamination.

**Table:** Sodium hypochlorite solution

Reagent	Final concentration	Amount
Sodium hypochlorite (NaClO)	5% v/v	1 mL
ddH_2_O	n/a	19 mL
**Total**	**n/a**	**20 mL**

Prepare the working solution on the day for sample processing.

**Table:** Bleach solution

Reagent	Final concentration	Amount
Bleach	10% v/v	2 mL
10% Triton X-100	0.01% v/v	20 μL
ddH_2_O	n/a	18 mL
**Total**	**n/a**	**20 mL**

Prepare the working solution on the day for sample processing.

**Table:** HgCl_2_ solution

Reagent	Final concentration	Amount
HgCl_2_	0.1%	0.1 g
ddH_2_O	n/a	100 mL
**Total**	**n/a**	**100 mL**

Prepare the solution on the day for sample processing.

**Table:** 1/2 MS horizontal plates containing 1/2 MS nutrients, 1% sucrose, pH5.7

Reagent	Final concentration	Amount
Murashige & Skoog (MS) Basal Salt Mixture	n/a	2.17 g
Sucrose	1 %	10 g
Phytagel	0.3%	3 g
ddH_2_O	n/a	Up to 1 L
**Total**	**n/a**	**1 L**

Adjust pH to 5.7 with 0.5 M KOH, and autoclave at 121°C for at least 15 min. Cool to ∼60°C and pour into disposable Petri dish after autoclave. Store MS plates at 4°C–8°C for up to 3 months.

***Alternatives:*** Phytagel (3 g) could be changed to agar (7 g) for 1/2 MS horizontal plates with 0.7% agar.

**Table:** 1/2 MS vertical plates containing 1/2 MS nutrients, 1% sucrose, pH5.7

Reagent	Final concentration	Amount
Murashige & Skoog (MS) Basal Salt Mixture	n/a	2.17 g
Sucrose	1%	10 g
Phytagel	0.6%	6 g
ddH_2_O	n/a	Up to 1 L
**Total**	**n/a**	**1 L**

Adjust pH to 5.7 with 0.5 M KOH, and autoclave at 121°C for at least 15 min. Cool to ∼60°C and pour into disposable Petri dish after autoclave. Store MS plates at 4°C–8°C for up to 3 months.

***Alternatives:*** Phytagel (6 g) could be changed to agar (12 g) for 1/2 MS vertical plates with 1.2% agar.

**Table:** 1/2 MS horizontal plates containing 1/2 MS nutrients, 25 mg/L cefotaxime, pH5.7

Reagent	Final concentration	Amount
Murashige & Skoog (MS) Basal Salt Mixture	n/a	2.17 g
Phytagel	0.3%	3 g
Cefotaxime stock solution, 25 mg/mL**∗**	25 mg/L	1 mL
ddH_2_O	n/a	Up to 1 L
**Total**	**n/a**	**1 L**

Adjust pH to 5.7 with 0.5 M KOH, and autoclave at 121°C for at least 15 min.

**∗*Note:*** After autoclave and cooled to ∼60°C, add cefotaxime stock solution and mix thoroughly, then pour into disposable Petri dish. Store MS plates at 4°C–8°C for up to 3 months.

***Alternatives:*** Phytagel (3 g) could be changed to agar (7 g) for 1/2 MS horizontal plates with 0.7% agar.

**Table:** Coelenterazine h stock solution

Reagent	Final concentration	Amount
Coelenterazine h	2 mM	0.815 mg
Ethanol	n/a	1 mL
**Total**	**n/a**	**1 mL**

Protect against exposure to light and store at −80°C for up to 6 months.

**Table:** Coelenterazine h working solution

Reagent	Final concentration	Amount
Coelenterazine h stock solution, 2 mM	10 μM	100 μL
10% Triton X-100	0.1% v/v	200 μL
ddH_2_O	n/a	19.7 mL
**Total**	**n/a**	**20 mL**

Prepare the working solution on the day for sample processing.

**Table:** Mannitol solution

Reagent	Final concentration	Amount
Mannitol	600 mM	54.65 g
ddH_2_O	n/a	500 mL
**Total**	**n/a**	**500 mL**

Store at 4°C for up to 1 month.

**Table:** NaCl solution

Reagent	Final concentration	Amount
NaCl	100 mM	2.92 g
ddH_2_O	n/a	500 mL
**Total**	**n/a**	**500 mL**

Store at room temperature (20°C–25°C) for up to 1 month.

**Table:** H_2_O_2_ solution

Reagent	Final concentration	Amount
30% H_2_O_2_	10 mM	0.511 mL
ddH_2_O	n/a	495 mL
**Total**	**n/a**	**500 mL**

Prepare the working solution on the day for sample processing.

**Table:** Discharging buffer

Reagent	Final concentration	Amount
CaCl_2_.2H_2_O	2 M	147 g
Ethanol	20% v/v	100 mL
ddH_2_O	n/a	364 mL
**Total**	**n/a**	**500 mL**

Store at room temperature (20°C–25°C) for up to 1 month.

**Table:** Cefotaxime stock solution

Reagent	Final concentration	Amount
Cefotaxime	25 mg/mL	250 mg
ddH_2_O	n/a	10 mL
**Total**	**n/a**	**10 mL**

Store at −20°C for up to 6 months.

**Table:** Cefotaxime working solution

Reagent	Final concentration	Amount
Cefotaxime stock solution, 25 mg/mL	25 mg/L	20 μL
ddH_2_O	n/a	20 mL
**Total**	**n/a**	**20 mL**

Prepare the working solution on the day for sample processing.

## Step-by-step method details

Screening EMS AEQ*sig6* libraries for mutants with reduced Ca^2+^ signal response

### Preparing plant materials for the first round of screening

**Timing: 2**–**4 h**

The purpose of these steps is to prepare plant materials for analyzing Ca^2+^ signal response.

1. Sterilize the M2 seeds with 5% sodium hypochlorite for 10 min in a 1.5 mL tube (one tube for each pool).
2. Rinse seeds in sterile-deionized water for 4 times.
3. Plant seeds evenly on a 150 mm × 15 mm round disposable Petri dish with a 0.9–1 cm interval (about 200 seeds per dish) on 0.3% Phytagel containing 1/2 MS nutrients, 1% sucrose, pH5.7 (one plate for each pool).
4. Seal plates with micropore tape (3M).
5. Stratify the seeds at 4°C–8°C for 3 days in the dark.
6. Grow seedlings horizontally in a Percival CU36L5 incubator at 21°C–23°C under a 16-h light, 8-h dark photoperiod for 9 days.

**CRITICAL:** Excessive light in the growth chamber and excessive sucrose in growth media should be avoided because the stressed seedlings are not suitable for monitoring Ca^2+^ signal response. Coelenterazines are very sensitive to oxidation and are used as a chemiluminescent indicator of ROS accumulation (Dubuisson et al., 2000). To avoid resulting problems, seedlings must be grown under optimal growth conditions, and aequorin should be reconstituted in the dark. We recommend screening 10–20 plates per day for skilled researchers.

### Analyzing transient Ca^2+^ signal response

**Timing: 6**–**9 h**

The purpose of these steps is to monitor the Ca^2+^ signal response of M2 AEQ*sig6* seedlings for screening mutants.

7. Draw a big arrowhead on the back of the plate to orientate the plate.
8. Spray the 9-day-old seedlings evenly with ∼2–3 mL/per dish of coelenterazine working solution (NanoLight, final concentration 10 μM in sterile dH_2_O, 0.1% Triton X-100 with a laryngeal or nasal spray).

**CRITICAL:** Apoaequorin binds to a prosthetic group, coelenterazine, to be converted to aequorin that reacts with Ca^2+^ (Shimomura et al., 1962). In the presence of Ca^2+^, coelenterazine is decomposed into coelenteramide and carbon dioxide with the emission of blue light at approximately 465 nm (Mithofer and Mazars, 2002). Coelenterazines are stable in solid form when stored at −80°C under nitrogen or argon, but unstable under light or in an aqueous solution because they are prone to be oxidized. The stock solution (2 mM in ethanol) should be prepared in ethanol or methanol and stored at −80°C in light-tight tubes and handled in the dark. Do not use DMF or DMSO to prepare stock solution because these solvents cause oxidation of coelenterazine. Use freshly prepared working solution.

9. Incubate Petri dishes for 4–6 h at room temperature (20°C–25°C) in the dark for aequorin reconstitution.
10. Pre-cool down the operating temperature of the CCD camera to −100°C.
11. Transfer the Petri dish to the light-tight box of the CCD imaging apparatus in the dark.

**CRITICAL:** To prevent chlorophyll autofluorescence from interfering with the luminescence recording, do not expose the seedlings to light after applied coelenterazine. If the seedlings are occasionally exposed, keep in the dark for 5 min to minimize chlorophyll autofluorescence.

12. Treat the seedlings with 600 mM mannitol in water (30 mL per 150 mm × 15 mm Petri dish) in the dark.

***Note:*** The solution of given stimuli should submerge the seedlings, but not too much.

13. Acquire the luminescence image (Image1) continuously for 2–5 min, immediately after treatment.
14. Discard the mannitol solution.
15. Place the Petri dish back to the light-tight box of the CCD imaging apparatus as previously orientated.
16. Treat the seedlings with 10 mM H_2_O_2_ in water (30 mL per 150 mm × 15 mm Petri dish).
17. Acquire the luminescence image (Image2) continuously for 2–3 min immediately after treatment.
18. Compare the Image1 with the Image2.
19. Pick up seedlings showed weaker luminescence in Image1 and normal luminescence in Image2 (about 0.5% of the total seedlings).

***Note:*** We assumed that most mutations do not affect the Ca^2+^ signal. Thus the majority of the M2 seedlings are considered as wild-type controls.

20. Rinse the mutant candidates with 25 mg/L cefotaxime solution in sterile-deionized water 2 times.

***Note:*** The use of cefotaxime reduces contamination by micro-organisms (Mittal et al., 2020).

21. Transfer the mutant candidates to 1/2 MS medium (without sucrose) with 25 mg/L cefotaxime under low light for recovery.

***Note:*** Dissolve cefotaxime in 1/2 MS medium. This step is not necessary for stimuli that do not cause severe damage.

22. Transfer the mutant candidates to the soil after 1–2 days recovery, and grow seedlings under optimal growth conditions.

**Figure 1:**
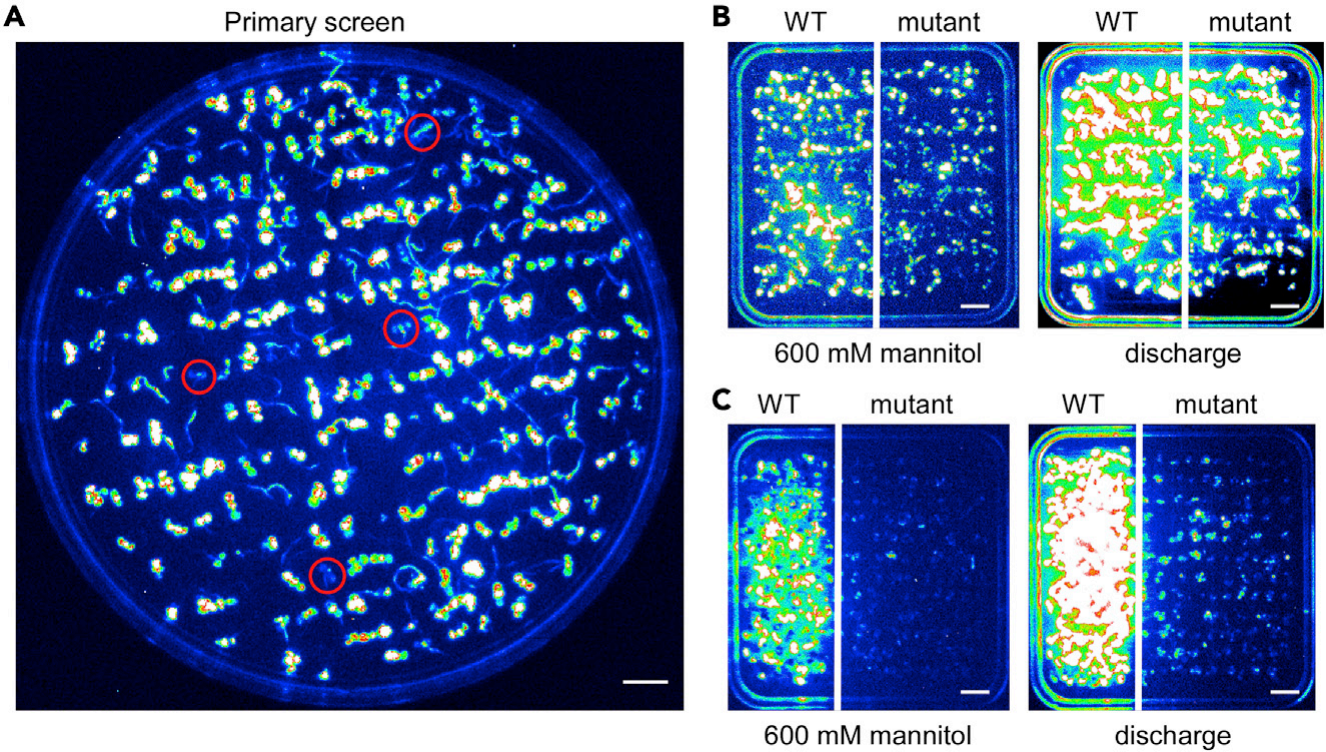
Identification of mutant candidates with reduced Ca^2+^ signal response (A) Isolation of putative candidates with reduced osmotic stress-triggered Ca^2+^ luminescence response in the first-round screen. The seedlings with red circles are the candidates. (B) Mutant candidate with reduced osmotic stress-induced Ca^2+^ luminescence response (left), but with similar discharged Ca^2+^ luminescence intensity (right). (C) A false mutant candidate with reduced osmotic stress-induced Ca^2+^ luminescence response (left), but with reduced discharged Ca^2+^ luminescence intensity (right). Scale bars, 1 cm.

**CRITICAL:** Steps 14–18 are optimal and should be carefully designed. Although these steps reduce the false positive mutant candidates, they may also have mutant candidates escaped. Some stimuli may share similar receptors or upstream regulators (Laohavisit et al., 2020, Wu et al., 2020). The alternative strategy is to pick up about 1.5%–2% of the total seedlings only according to Image1 (Figure 1A). The recommended ranges for stimuli were listed in Table 2.

**Pause point:** Seeds can be safely stored at dry conditions at room temperature (20°C–25°C) for 2–5 years. As an alternative, seeds can be safely stored at −20°C for more than 5 years with high viability.

### Second- to fourth-round screens

**Timing: 2 weeks**

The purpose of these steps is to confirm the defective Ca^2+^ signal response of these mutant candidates isolated during the first-round screen.

23. Sterilize the seeds of mutant candidates and the wild type AEQ*sig6*, as steps 1 and 2.
24. Plant seeds evenly on a 90 mm × 15 mm round or 100 mm × 15 mm square disposable Petri dish with a 0.9–1 cm interval on 0.3% Phytagel containing 1/2 MS nutrients, 1% sucrose, pH5.7 (at least three plates for each line). Prepare plant materials as steps 4–6.
25. Treat the 9-day-old seedlings with 600 mM mannitol, 10 mM H_2_O_2_ or other stimuli, as steps 7–13.
26. Acquire the luminescence image (Image3) continuously for 2–5 min, immediately after treatments.
27. Discard the mannitol solution and place the Petri dish back to the light-tight box of the CCD, as steps 14 and 15.
28. Treat the seedlings with discharging buffer (2 M CaCl_2_ in 20% ethanol).

***Note:*** The high concentration of ethanol destroys the cell membrane, allowing Ca^2+^ to penetrate the cells and discharge the remaining aequorin.

29. Acquire the luminescence image (Image4) continuously for 5 min, immediately after treatments.
30. Mutant candidates with reduced luminescence in Image3, and standard or enhanced luminescence in Image4 are preferable for third- to fourth- round screens (Figure 1B).
31. Select candidate mutants with reduced Ca^2+^ signal for third- to fourth-round screens and transfer the mutant plant to soil and harvest seeds from 8 individual seedlings using the single pedigree-based seed collection to obtain the homozygous, in case of a dominant mutation.
32. Analyze phenotypes of mutant candidates under osmotic stress. It should be aware that stimuli responses are controlled both by Ca^2+^-dependent and -independent processes (Kadota et al., 2014, Li et al., 2014, Chen et al., 2020, Yuan et al., 2014).

### Screening AEQ*ub* EMS library for altered Ca^2+^ signal responsive mutants using the film adhesive seedling (FAS) system

#### Preparing plant materials for the first-round screen

**Timing: 12 days**

The purpose of these steps is to monitor the Ca^2+^ signal of M2 AEQ*ub* seedlings.

33. **Prepare M2 AEQ*ub* seedlings for luminescence imaging.** Sterilize EMS-mutagenized M2 seeds of AEQ*ub* plants with a 10% bleach solution containing 0.01% Triton X-100 or 0.1% HgCl_2_ for 5 min and rinse with sterile water 4–5 times. Sow the sterilized seeds on a square plate (10 × 10 cm square Petri dish with grid) containing 1/2 MS, 1% sucrose, and 1.2% agar. Place plates vertically in a growth chamber for 7 days, after stratification at 4°C for 2 days.  
34. **Transfer the seedlings onto a film.** Place an adhesive film (Thermo Scientific Adhesive PCR Plate Seals) on the top of 7-day-old seedlings growing on the vertical plate. Gently push the film by hand to ensure that seedlings adhere to the film, and peel the film gently to let the seedlings adhered to the film (Figures 2A–2C).

**Figure 2:**
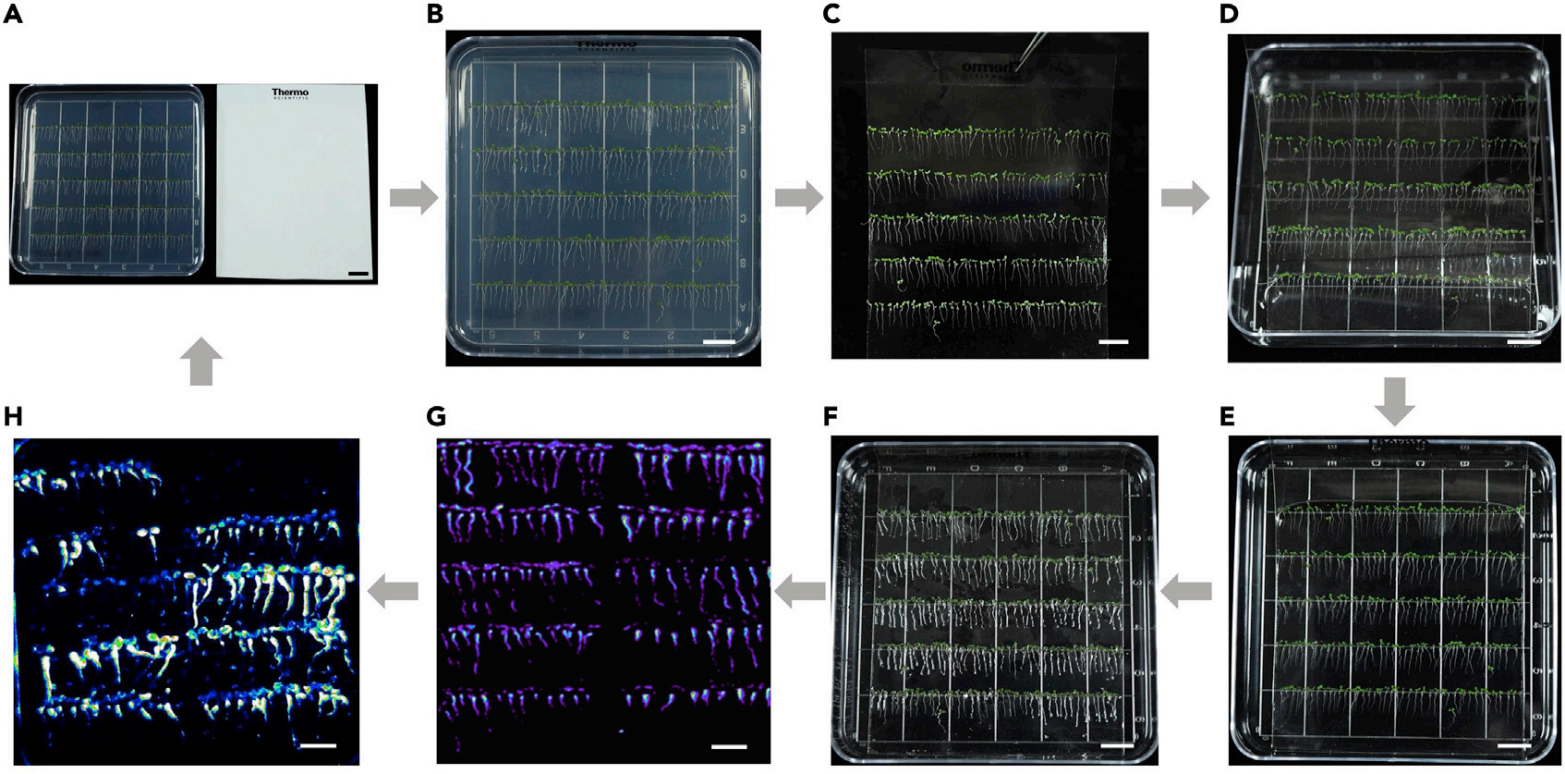
The workflow of EMS AEQ*ub* library screening for the mutants with altered Ca^2+^ response using the FAS system (A) Prepare a square plate with 7-day-old seedlings (left) and an adhesive film (right). (B) Place the film on the top of seedlings and gently touch the film to have seedlings adhered to. (C–E) Peel the film (C) and place it into the plate containing coelenterazine solution with the seedlings side down (D) to have seedlings co-incubated with coelenterazine overnight (9–11 h) at room temperature (20°C–25°C) with gently shaking (E). (F) Discard the coelenterazine solution and place the film back to the plate with the seedling side up. (G and H) Acquire Ca^2+^ luminescence images for the first-round of the screen (G) and the second-round of verification (H). Scale bars, 1 cm.
35. **Incubate the seedlings with coelenterazine.** Place the adhered seedlings onto the square plate (10 × 10 cm) containing 15 mL of 1 μg/mL h-coelenterazine (15 μl of 1 mg/mL coelenterazine added to 15 mL dH_2_O). Incubate the seedlings at room temperature (20°C–25°C) for 4 h to overnight (9–11 h) (Figures 2D and 2E).
36. **Prepare for luminescence imaging.** Discard the h-coelenterazine solution and place the film with seedlings face-up within the same plate. Leave the plate in the dark for 5 min (Figure 2F).
37. **Acquire luminescence images.** In the dark, place the plate on the stage of the CCD imaging system. Acquire images immediately and continuously for 2–5 min upon adding 20 mL of stimuli solution to the plates (Figures 2G and 2H). The concentrations of stimuli were listed in Table 2.
38. **Analyze luminescence images and pickup mutant candidates.** Set the same display range for all acquired luminescence images. Pick the seedling with altered Ca^2+^ luminescence, let them recover on 1/2 MS medium (without sucrose) with 25 mg/L cefotaxime for two days before transferring to soil.

### Second- to fourth-round screens

**Timing: 2 weeks**

The purpose of these steps is to confirm the mutant candidates isolated during the first-round screen.

**Figure 3:**
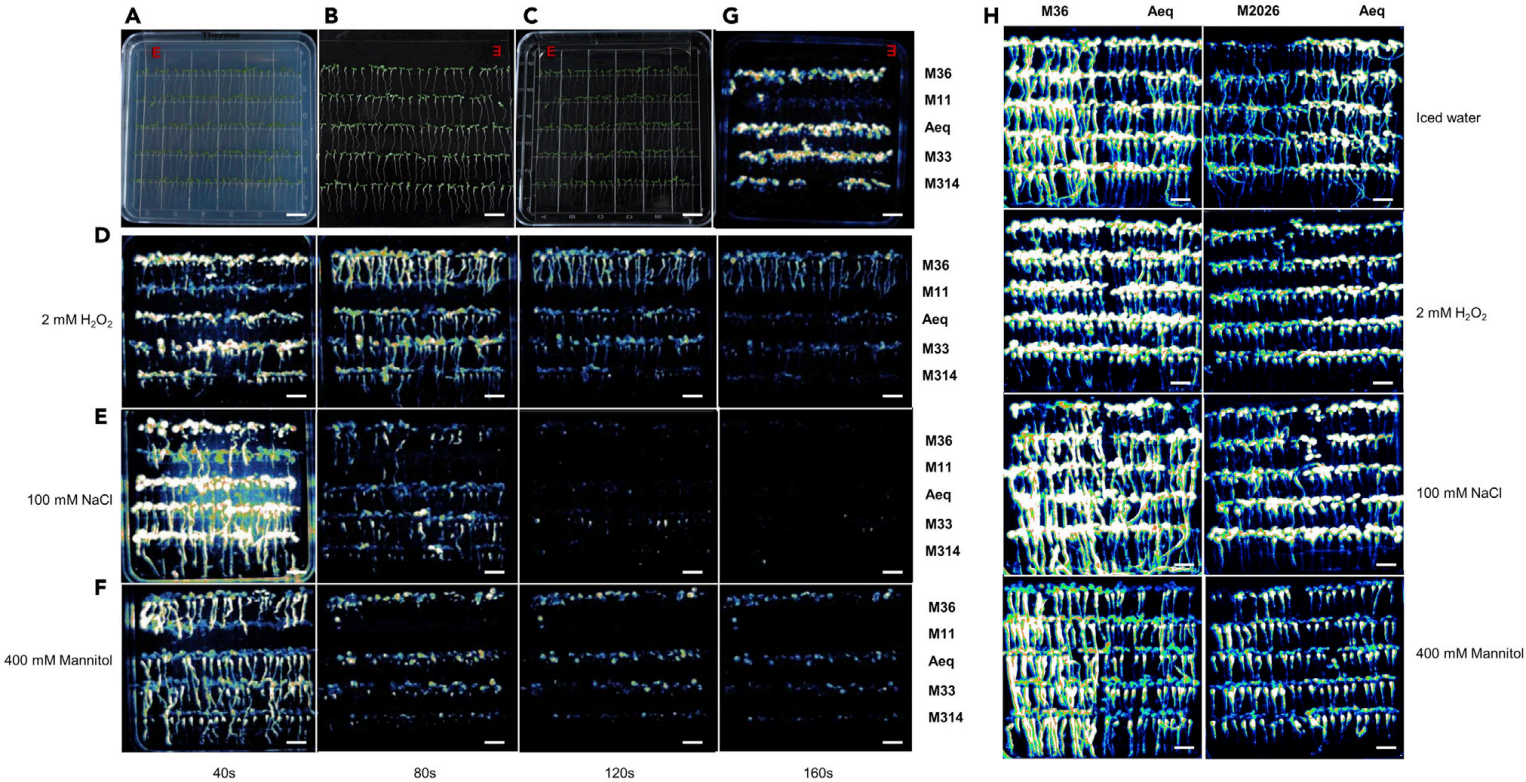
Verification of mutant candidates (A–C) Prepare mutant candidates for the second-round of FAS Ca^2+^ imaging as described above. Red-letter E is marked for the orientation of the film. The seedlings of each mutant together with wild type AEQ*ub* were grown in one line. (D–F) Second-round of FAS Ca^2+^ imaging for mutant verification. Acquire H_2_O_2_ (D), NaCl (E), and mannitol (F) induced Ca^2+^ luminescence images for 160 s at an interval of 40 s. (G) Acquire discharged images. Discard the stimuli and acquire images for one minute right after applying the discharge solution to stimuli-treated films. (H) The third round of FAS Ca^2+^ imaging for mutant verification. Mutant candidates and wild-type control were grown on the same plate and transferred onto the same film for FAS Ca^2+^ imaging. Altered Ca^2+^ luminescence responses of the mutant were recorded under cold, oxidative, salt, and osmotic stress as compared to wild type. Scale bars, 1 cm.
40. Acquire images at an interval of 40 s for 160 s immediately upon adding 20 mL of 100 mM NaCl, 400 mM mannitol, 1 mM H_2_O_2_ to each plate, respectively (Figures 3D–3F). Acquire discharged images by applying 20 mL of discharge solution (2 M CaCl_2_ in 20% ethanol) to the plate containing the previous stimuli-treated seedlings (Figure 3G).
41. Analyze luminescence images by comparing Ca^2+^ signal response images to its discharge images of mutant candidates. Mutant candidates with enhanced or reduced Ca^2+^ luminescence to given stimuli, but with similar luminescence intensity of discharged images should be considered for further verification by performing third- to fourth-round confirmation (Figure 3H).

***Note:*** As noted above, the physiological status of seedling significantly affects Ca^2+^ signal response. To avoid the interference by defect of seed germination and growth, we recommend using the different batches of mutant seeds together with their wild-type AEQ*sig6* or AEQ*ub* that were harvested under the same growth condition for each round screen.

39. Sterilize the seeds of mutant candidates and their wild-type AEQ*ub* and grow the seedlings on a square plate as indicated in Figures 3A–3C. Prepare four replicates of the same orientated seedlings for FAS imaging as steps 33–36.

**CRITICAL:** Mutant candidates with reduced luminescence intensity of discharged aequorin should be eliminated (Figure 1C and mutant 11 in Figures 3D–3G). These could be caused by reduced expression of the *apoaequorin* gene due to mutations or silencing, or defects in coelenterazine absorption or aequorin reconstitution as described above.

### Quantitative Ca^2+^ measurement using a luminometer with AEQ seedlings

**Timing: 10 h**

The purpose of these steps is to determine the Ca^2+^ signal dynamical features of mutants in response to given stimuli.

42. Place a single 7-day-old AEQ*sig6* or AEQ*ub* seedling into a 1.5 mL Eppendorf tube that contains 1 mL of 1 μg/mL h-coelenterazine and place them in a light-seal box and leave it on a shaker (80 rpm/min) at room temperature overnight (20°C–25°C, 9–11 h).
43. Place the tube contains the seedling into the luminometer chamber. Acquire the signal for 60 s at 1 s intervals for resting luminescence.
44. Acquire luminescence intensity (L_stimuli_) at 1 s intervals for 80–120 s (depended on the duration of Ca^2+^ luminescence response) immediately after adding 1 mL of 400 mM mannitol, or 100 mM NaCl, or 5 mM H_2_O_2_ solution into the tube.
45. Discard the stimuli solution, and acquire the luminescence intensity (L_rest_) at 1 s intervals about 2 min until values were within 5% of the highest discharged value, immediately after adding 1 mL of discharging buffer (2 M CaCl_2_ in 20% ethanol).

***Note:*** Steps 42–45 can be adjusted depending on the type of luminometer used (Mittal et al., 2020, Tanaka et al., 2013).

**Figure 4:**
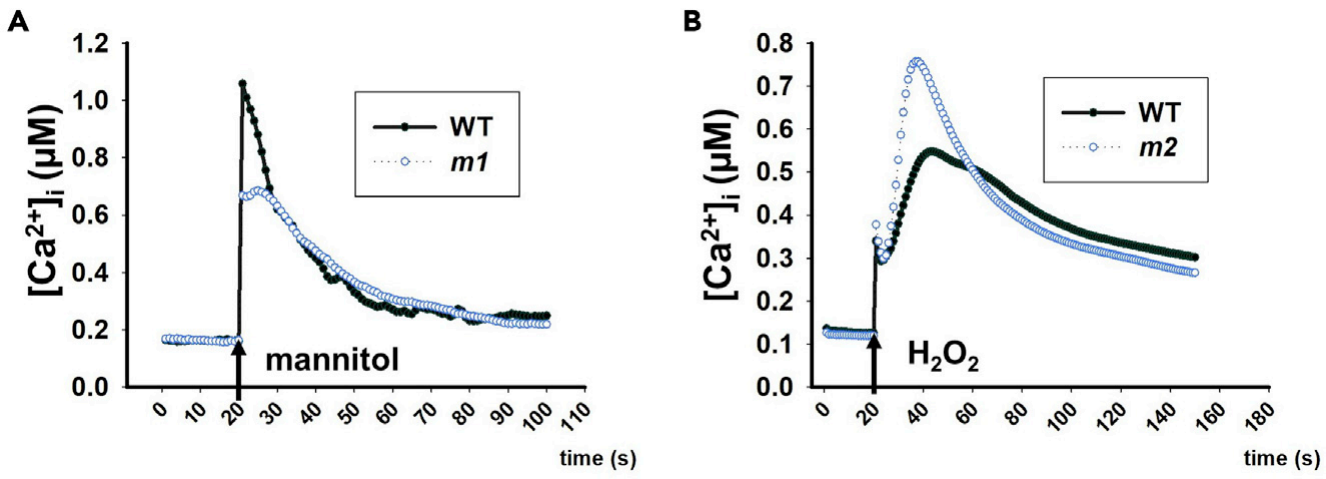
Quantification of cytosolic Ca^2+^ elevation in mutant candidates with altered Ca^2+^ signal responses using a luminometer (A) Quantification of cytosolic Ca^2+^ elevation in WT and *m1* after treated with 400 mM mannitol. (B) Quantification of cytosolic Ca^2+^ elevation in WT and *m2* after treated with 5 mM H_2_O_2_. Arrows indicate the time points for treatments.

46. The total luminescence (L_max_) is calculated by L_rest_ plus L_stimuli_. The calibration equation is adopted from the previously described equation (Knight et al., 1996): pCa = 0.332588 (-log*k*) + 5.5593, where *k* is a rate constant equal to stimuli triggered aequorin luminescence (L_stimuli_) divided by total luminescence (L_max_). Mutants with either altered amplitudes or durations are shown in Figure 4.

### Whole-genome resequencing

**Timing: 10 months**

The purpose of these steps is to clone the gene with the mutation responsible for the altered Ca^2+^ signal response.

47. Backcross the mutant with wild-type AEQ*sig6* or AEQ*ub*, and analyze the mannitol-induced Ca^2+^ signal in F1 seedlings. When F1 shows a wild-type phenotype, it is a recessive mutation, and vice versa, it is a dominant mutation. We suppose the mutation is recessive here.
48. Generate the backcrossed F2 population, the ratio of mutant-like and WT-like seedlings is 1:3 in the backcrossed F2 population.
49. Analyze the mannitol-induced Ca^2+^ signal response in the F2 population as steps 1–22, and pick up putative mutant seedlings with reduced mannitol-induced Ca^2+^ luminescence response. At least 200 F2 seedlings were selected and planted in soil. Collect the DNA of each candidate F2 mutants.
50. Harvest F3 seeds from single F2 seedlings.
51. Analyze the mannitol-induced Ca^2+^ signal in the F3 seedlings.
52. Select 40–50 single lines with reduced Ca^2+^ luminescence, and pooled the corresponding previously collected F2 DNA together for whole-genome resequencing (Abe et al., 2012).
53. Resequence the mutant parent line and the wild-type AEQ*sig6.*
54. Look for SNP changes only present in the mutant bulk as described previously (Abe et al., 2012).

***Alternatives:*** Map-based cloning using Wassilewskija (Ws) background also works (Yuan et al., 2014), but will also require a larger population to be screened.

## Expected outcomes

The mutants with reduced or enhanced Ca^2+^ signal response to osmotic stress or given stimuli as described could be isolated. These two systems are designed to identify receptors, sensors, or upstream regulators in signaling pathways that involve Ca^2+^ response. We screened about 100,000 M2 seedlings, and about 567 mutant candidates were isolated from the initial screen using the AEQ*sig6* EMS mutagenized library. Several mutants were selected for further analysis after second- to fourth-round screens, together with mutant growth phenotype analysis under osmotic or other abiotic stress treatments.

## Quantification and statistical analysis

All luminescence imaging data are captured and analyzed by Winview/32. We adjust an appropriate range of display layouts, which allows the visualization of luminescence signals from the majority of seedlings. Subtraction of the background signal from the raw signal resulted in the Ca^2+^ luminescence signal of individual seedlings.

Quantitative Ca^2+^ measurement using a luminometer is described in step 46. The detailed data processing is provided in Table S1, using the raw data for Figure 4B.

## Limitations

Although the aequorin-based Ca^2+^ luminescence system provides a viable platform to identify genes of interest in Ca^2+^ response-related signaling pathways, it still has the following limitations.

1) Plants display tissue- and stimuli-specific Ca^2+^ signal responses as described previously (Zhu et al., 2013). For example, H_2_O_2_, NaCl, cold and amino acids cause more substantial and robust Ca^2+^ luminescence in leaves. In contrast, mannitol-induced Ca^2+^ responses are relatively weaker in leaves, and somewhat more potent in roots. Tissue-specific Ca^2+^ signal responses make the isolation of osmotic responsive mutants more challenging and less efficient using the first protocol, but more accessible using the second protocol. Even performed by skilled researchers, achieving a more robust and reproducible Ca^2+^ luminescence response is still challenging.

2) Screening and cloning are labor-intensive and time-consuming and require researchers with well-trained skills, stable funding, expensive equipment, and enough space. Detailed experiments should be well designed in advance.

## Troubleshooting

### Problem 1

Weak Ca^2+^ luminescence response (steps 13, 17, 26, and 29).

### Potential solution

It is recommended to optimize the strength of stimuli for triggering Ca^2+^ luminescence response to screen either reduced or enhanced Ca^2+^ responsive mutants. Generally, for a screening of reduced Ca^2+^ responsive mutants, the use of high strength stimuli should be considered. In contrast, for a screening of enhanced Ca^2+^ responsive mutants, the low strength of stimuli should be applied. The weak Ca^2+^ luminescence response could be caused: 1) the suboptimal concentration and application method of stimuli; 2) the suboptimal concentration and co-incubation condition of coelenterazine; 3) the oxidation of coelenterazine that makes screening less effective; 4) the seedlings are too young or too old, or suffer from stress before imaged; 5) the CCD camera system is not on optimal work conditions. Therefore, the strength of any given stimuli should be optimized to meet the purpose of screening. The concentration of coelenterazine and stimuli mentioned in the protocol should be adjusted based on the intensity of luminescence and increased or decreased if luminescence response is too weak or too strong.

### Problem 2

Uneven Ca^2+^ luminescence responses (steps 13, 17, 26, and 29).

### Potential solution

It is recommended that EMS seeds are sieved through 0.25 mm mesh before planting on the plate. AEQ*sig6* seeds are planted evenly on a 150 mm × 15 mm round disposable Petri dish with a 0.9–1 cm interval with about 200 seeds per dish (Figure 1). AEQ*ub* seeds are planted as 5 lines along the grid on a 10 × 10 cm square Petri dish with ∼200 seeds per dish (Figure 2). Grow plates on the optimal conditions to have uniform seedlings grown well on the plate and adhered to the film. FAS system allows a co-incubation of uniform seedlings adhered to the same film with coelenterazine to avoid the variations due to uneven spraying of coelenterazine. Therefore, the FAS system offers a more sensitive, reproducible, and reliable platform for monitoring Ca^2+^ luminescence response as the advised performances were followed.

### Problem 3

High noise (steps 13, 17, 26, and 29)

### Potential solution

The optimal working temperature for the CCD camera is −100°C. Inappropriate working temperature leads to the strips-like noise signal on the full image. The CCD imaging system should be placed in a dark room to avoid visible light signal interference, which can cause dots-like noise.

### Problem 4

Lose focus (steps 13, 17, 26, and 29)

### Potential solution

Adjust the focus of the CCD camera to the seedlings on the sample stage, with the door of the box open under weak light (such as the light of the computer screen).

### Problem 5

Moisture in CCD camera (steps 13, 17, 26, and 29)

### Potential solution

Moisture tends to make images blurry and reduce the overall life span of the CCD camera. Thus it is crucial to maintain a low humidity condition and clean the camera regularly. The most effective way to remove moisture is using silica gel in the light-tight box and keeping the box closed.

### Problem 6

Candidate mutant cannot be rescued (steps 19–22).

### Potential solution

It is recommended to use a low strength of stimuli and immediately rinse the seedlings of mutant candidates two times to reduce damage caused by stimuli, and place mutant candidates on 1/2 MS medium with 25 mg/L cefotaxime to avoid contamination by micro-organisms (Mittal et al., 2020).

### Problem 7

Candidate mutant does not express aequorin (step 29).

### Potential solution

Although T-DNA insertion mutagenesis makes the cloning process more accessible, we recommended using EMS mutagenesis rather than T-DNA insertion mutagenesis to generate the screening populations. When transformed AEQ*sig6* plants with transgenes, apoaequorin is occasionally silenced, most likely due to siRNA-mediated transcriptional silencing (Mlotshwa et al., 2010).

### Problem 8

Seedlings float after liquid treatment (steps 12, 16, 25, and 28)

### Potential solution

The first protocol is used to screen mutants with seedlings growing on horizontal MS-containing agar plates. Try to adjust agar concentration for the root to get into the solid medium to solve this problem. The MS medium with 0.3% Phytagel or 0.7% agar is recommended.

## Resource availability

### Lead contact

Further information and requests for resources and reagents should be directed to and will be fulfilled by the lead contact, Yang Zhao (yangzhao@psc.ac.cn).

### Materials availability

All wild-type plant materials, including AEQ*sig6* and AEQ*ub*, described in this study are available upon request.

### Data and code availability

This study did not generate/analyze datasets.

## Supplemental information

Table S1. Detailed data processing of Ca^2+^ signals, related to step 46
